# Conformally Gated Surface Conducting Behaviors of Single-Walled Carbon Nanotube Thin-Film-Transistors

**DOI:** 10.3390/ma14123361

**Published:** 2021-06-17

**Authors:** Kyung-Tae Kim, Keon Woo Lee, Sanghee Moon, Joon Bee Park, Chan-Yong Park, Seung-Ji Nam, Jaehyun Kim, Myoung-Jae Lee, Jae Sang Heo, Sung Kyu Park

**Affiliations:** 1 Department of Electrical and Electronics Engineering, Chung-Ang University, Seoul 06974, Korea; ktkim0314@gmail.com (K.-T.K.); lkw9941@naver.com (K.W.L.); mshhwaa@gmail.com (S.M.); wnsbee@gmail.com (J.B.P.); mgs01170@gmail.com (C.-Y.P.); seungjieee@gmail.com (S.-J.N.); 2 Department of Chemistry and Materials Research Center, Northwestern University, 2145 Sheridan Road, Evanston, IL 60208, USA; jaehyun@northwestern.edu; 3 Convergence Research Institute, Daegu Gyeongbuk Institute of Science and Technology (DGIST), Daegu 42988, Korea; myoungjae.lee@dgist.ac.kr; 4 School of Advanced Materials Science and Engineering, Sungkyunkwan University, Suwon 16419, Korea

**Keywords:** single-walled carbon nanotube (SWCNTs), high purity SWCNT separation process, thin-film transistors (TFTs)

## Abstract

Semiconducting single-walled carbon nanotubes (s-SWCNTs) have gathered significant interest in various emerging electronics due to their outstanding electrical and mechanical properties. Although large-area and low-cost fabrication of s-SWCNT field effect transistors (FETs) can be easily achieved via solution processing, the electrical performance of the solution-based s-SWCNT FETs is often limited by the charge transport in the s-SWCNT networks and interface between the s-SWCNT and the dielectrics depending on both s-SWCNT solution synthesis and device architecture. Here, we investigate the surface and interfacial electro-chemical behaviors of s-SWCNTs. In addition, we propose a cost-effective and straightforward process capable of minimizing polymers bound to s-SWCNT surfaces acting as an interfering element for the charge carrier transport via a heat-assisted purification (HAP). With the HAP treated s-SWCNTs, we introduced conformal dielectric configuration for s-SWCNT FETs, which are explored by a carefully designed wide array of electrical and chemical characterizations with finite-element analysis (FEA) computer simulation. For more favorable gate-field-induced surface and interfacial behaviors of s-SWCNT, we implemented conformally gated highly capacitive s-SWCNT FETs with ion-gel dielectrics, demonstrating field-effect mobility of ~8.19 cm^2^/V⋅s and on/off current ratio of ~10^5^ along with negligible hysteresis.

## 1. Introduction

Recently, non-conventional semiconducting channel layers provided by low-dimensional unit structures such as nanotubular networks or quantum dots have emerged as promising candidates for next generation electronic applications [1,2,3,4]. Particularly, nanotubular linked single-walled carbon nanotubes (SWCNTs) have consistently attracted attention in a variety of electronic applications due to their outstanding electrical and mechanical characteristics such as high carrier mobility, good chemical and thermal stability, and excellent mechanical durability [5,6]. Moreover, the SWCNT film can be fabricated via a simple solution process, which can enable their utilization in cost-effective and large-area applications [7,8]. Despite intensive research on SWCNT-based electronic devices, they often suffer from their distinct geometric features such as less flattened and unsmooth surface linkages of the nanotubes, exhibiting limited electrical characteristics possibly due to non-uniform electric field distribution and resistive interconnection between the nanotubes. Typically, SWCNTs are able to be adapted as a transparent electrode and a semiconductor depending on the degree of separation of metallic (m-) nanotubes from semiconducting (s-) nanotubes during dispersion because most as-synthesized SWCNTs have the coexistence of m- and s-SWCNTs [9,10,11]. Therefore, for the specific applications of SWCNTs to semiconducting channels of various electronics, high purity s-SWCNTs should be efficiently and separately collected by an appropriate dispersion of SWCNTs. Recently, as an effective strategy for the functionalization of s-SWCNTs preserving their intrinsic characteristics, non-covalently modified s-SWCNTs with conjugated polymers have remarkably been developed due to their simplicity, high selectivity, and high yield [12,13,14]. Although such conjugated polymers have been found to efficiently separate s-SWCNT from m-SWCNT, however, there is still some problematic issues on the electrical performances of the s-SWCNT-based electronics such as limited electric field-induced charge carrier distribution on the semiconductor surface and charge carrying ability between the s-SWCNT nanotubular networks. Typically, the conjugated polymers bonded with s-SWCNTs often impede to induce and carry the charge carriers on the nanotubular channel surface, which causes degradation of electrical properties [15,16,17]. Therefore, it is necessary to remove the conjugated polymers that interact with s-SWCNTs after the dispersion of s-SWCNTs to achieve desirable performances as a semiconductor material. Previously, several approaches have been employed to remove these polymers from s-SWCNTs using extensive washing and high-power sonication/ultra-centrifugation processes [18,19]. Despite its high efficiency in purifying and sorting the s-SWCNTs, the process is rather complex and costly. In addition to the separation of s-SWCNT, for high-performance SWCNT-FETs, the interface surface between the semiconductor and gate dielectric layer should be considered as a crucial factor because of 1D-randomly distributed rough configuration of SWCNTs that negatively influences electrical performances of FETs. For example, in the case of the conventional bottom-gate and top-contact SWCNT FETs, the s-SWCNT channel layer is placed on a gate dielectric layer and structurally exposed in ambient species in air, causing unstable SWCNT FETs. Additionally, it is difficult to form an optimal and conformal contact between the SWCNT channel and gate dielectric layer, because the SWCNT network is inherently porous and has a rough surface [20,21,22]. In this regard, conformally gated soft dielectric materials such as organic and ionotronic materials may introduce more favorable device structures to achieve intimate contact with the nanotubular semiconducting networks overwhelming the vacuum deposited conventional inorganic dielectrics. According to that, more investigations for the surface conducting and interfacial behaviors of s-SWCNT FETs corresponding to structural integrity with the semiconducting nanotube networks and dielectric materials are needed to achieve highly conductive and stable s-SWCNT electronic devices.

Here, we report a facile process capable of minimizing interfering-polymers bound to the SWCNT surfaces for charge carrier transport between nanotubes during the dispersion of s-SWCNTs via a heat-assisted purification (HAP). The s-SWCNTs were obtained by selective dispersion with poly(3-dodecylthiophene) (P3DDT). After the dispersion of s-SWCNTs, a thermal treatment at 140 °C would induce the side-chain fluctuations of P3DDT in the P3DD-sorted s-SWCNTs solution, resulting in s-SWCNTs aggregation by prohibiting interaction between polymers and s-SWCNT. Then, with high purity s-SWCNTs obtained by re-dispersion of the s-SWCNTs aggregation, we demonstrate solution-processed s-SWCNT-FETs on the Al_2_O_3_ gate dielectric that exhibited good hole-carrier average mobility of 1.37 cm^2^·V^−1^·s^−1^ with on/off ratio of ~10^6^, compared to that without the HAP. The results imply that the P3DDT bound to s-SWCNTs can interfere with charge carrier transport between nanotubes despite the effective separation of s-SWCNT from m-SWCNT. Additionally, we investigate the influence of gate dielectric structural conformation (on- or in-dielectric) in the s-SWCNT-FETs on induced charge and trap density at the interface between the s-SWCNTs channel and organic/ion-gel dielectric layer. As a result, compared to on-dielectric configuration devices, devices with in-dielectric configuration showed improved electrical performances. This is attributed to conformal contact that can be formed in the in-dielectric structure by wholly covering rough surfaces of the s-SWCNT channel layer that acts as a defect at the interface. In particular, by applying finite-element analysis (FEA) simulation and various electrical characterizations, we evidently explore the device’s structural conformation for high performance and stable s-SWCNT FETs. Moreover, in order to further strengthen the impact of in-dielectric configuration in the s-SWCNT FETs, ion-gel gate dielectric was employed. As a result, ion-gel gated SWCNT FETs with more conformal contact even further accumulates charge carriers as well as reduces interface trap density so that their electrical characteristics are considerably enhanced, exhibiting the highest field-effect mobility of 8.19 cm^2^·V^−1^·s^−1^ with negligible hysteresis characteristics.

## 2. Materials and Methods

### 2.1. Preparation of High Purity s-SWCNT Solution

Polymer-wrapped SWCNT solution was prepared by mixing 5 mg of high-pressure CO (HiPCO) SWCNTs (diameter 0.8–1.2 nm, NanoIntegris, Boisbriand, QC, Canada) and 5 mg of regioregular poly(3-dodecylthiophene) (rr-P3DDT) in 25 mg of toluene. The solution was ultra-sonicated (700 W, 30%; Qsonica, Newtown, CT, USA) for 3 h at 50 °C in a bath. Then, the solution was centrifuged (Micro Centrifuge, Smart R17, Hanil Science Co., Ltd., Daejeon, Korea) at 15,000 rpm for 30 min and subsequently, a supernatant containing polymer wrapped s-SWCNTs was collected. Such centrifugation and collection processes were repeated for 3 times to remove SWCNT bundles and metallic species. Afterward, for the heat-assisted purification, 2 mL of the rr-P3DDT wrapped s-SWCNT solution was placed on a hot plate at 140 °C for 5 min. As a result, the s-SWCNTs are gradually agglomerated and precipitated at the bottom of the solution. Then, the aggregated s-SWCNT solution was collected, followed by the centrifugation at 15,000 rpm for 5 min. Finally, the purified s-SWCNT sediment was collected and re-dispersed by the ultra-sonication. Note that highly purified s-SWCNT is called a heat-assisted purification (HAP) treated s-SWCNT solution.

### 2.2. Preparation of Gate Dielectric Solutions (CYTOP and Ion-Gel)

CYTOP was prepared by diluting CYTOP solution (CTL-809M, Asahi Glass, Tokyo, Japan) and solvent (CT-solv.180, Asahi Glass, Tokyo, Japan) with a volume ratio of 3:1. For the ion-gel, the ion liquid 1-ethyl-3-methylimidazolium bis (trifluoro-methylsulfonyl) imide ([EMIM][TFSI]), poly (ethylene glycol) diacrylate), and photo initiator 2-hydroxy-2-methylpropiophenone were mixed with a weight ratio of 80:20:3. Then, this mixture was stirred at 80 °C and 1000 rpm for 6 h.

### 2.3. Fabrication of s-SWCNT FETs on Al_2_O_3_ Gate Dielectric

Heavily p-doped Si substrate was used as a bottom gate and an atomic-layer deposited (ALD) Al_2_O_3_ with a 60 nm of thickness was used as a gate dielectric layer. For a bottom contact device’s configuration, Cr (3 nm)/Au (30 nm) was deposited as source and drain (S/D) electrodes, on the Al_2_O_3_ gate dielectric and patterned by using a thermal evaporation and a lift-off process, respectively. Then, 40 μL of the HAP treated s-SWCNT solution was spin-coated on it and annealed at 160 °C for 1 h to evaporate toluene solvent. The s-SWCNT channel layer was patterned via both photolithography and dry-etching process with a reactive ion etching system (RIE, 100 W for 1 min, Daeki High-Tech. Co., Ltd., Daejeon, Korea).

### 2.4. Fabrication of a Side Gate High-Purity SWCNT FETs Using Ion-Gel Gate Dielectric

For demonstration of a coplanar-structured s-SWCNT FETs with ion-gel gate dielectric, 33 nm-thick Cr/Au was deposited simultaneously on a bare glass substrate as gate and S/D electrodes. Then, the s-SWCNT channel layer was formed and patterned by using spin-coating and the RIE process, respectively. After that, in order to fabricate a bank area for ion-gel gate dielectric layer, a negative photoresist (N-PR, SU-8 3005, Microchem Corp. Westborough, MA, USA) was used. Subsequently, ion-gel was drop-casted on the bank, followed by a photo-curing process with UV light and a photo-mask.

### 2.5. Characterization

The electrical characteristics of the s-SWCNT FETs were measured by using a semiconductor parameter analyzer (Agilent 4156C, Agilent Technologies, Santa Clara, CA, USA) in ambient air. The surface morphology and areal density of the SWCNT films were obtained using atomic force microscopy (AFM) (NX10, Park SYSTEMS, Suwon, Korea) with non-contact atomic force mode. Raman spectra were measured using a WITec confocal Raman microscope (alpha300R, WITec, Ulm, Germany) which is equipped with a piezo-scanner and an intensity-tunable 532 nm Nd:YAG laser (neodymium-doped yttrium aluminum garnet). UV-vis-NIR measurements of all SWCNT dispersions were carried out in quartz cells by using a spectrophotometer (Cary 5000, Agilent Technologies, Santa Clara, CA, USA). Note that all spectra for SWCNT solutions were recorded in the range of 400–1500 nm. C-V and C-F characteristics of gate dielectrics were investigated by using a metal-insulator-semiconductor (MIS; Au/Al_2_O_3_, CYTOP, or ion-gel/s-SWCNTs) and a metal-insulator-metal (MIM; Au/Al_2_O_3_, CYTOP, or ion-gel/Au) structure, respectively, with LCR meter (Agilent 4284A, Agilent Technologies, Santa Clara, CA, USA) (Figure S1).

## 3. Results and Discussion

**Figure 1:**
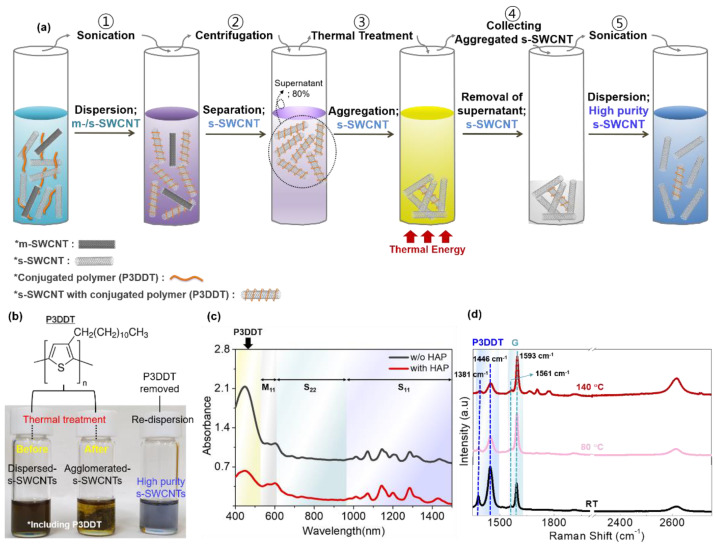
(**a**) High-purity s-SWCNT solution fabrication procedure; (**b**) Photographs of pristine, thermal treated pristine, and high-purity s-SWCNT solutions. The inset is a chemical structure of P3DDT; (**c**) Absorption spectra of the pristine and high-purity s-SWCNT; (**d**) Raman spectra of the pristine (black), 80 °C (pink), and 140 °C (red) temperature treated s-SWCNT at 532 nm excitation.

To prepare the s-SWCNT solution, high-pressure carbon monoxide (HiPCO)-synthesized SWCNTs and P3DDT were dispersed in toluene through sonication as shown in Figure 1a. In this step, the C_12_H_25_ side chains of P3DDT molecules bind to the surface SWCNTs by strong π-π interaction, followed by effective SWCNT dispersion (step 1) [23,24,25]. Afterward, the solution was centrifuged and approximately 80% of the supernatant was collected and residual solvent containing undispersed m-SWCNTs and SWCNT bundles was removed (step 2). This conventional purification and sorting route is definitely efficient in separating the s-SWCNTs from the undesired SWCNT mixture, however, a considerable amount of P3DDT molecules is still bound to s-SWCNTs which may disturb the carrier transport between the nanotubes. Therefore, it is essential to remove or reduce the amount of P3DDT molecules to improve the charge transport in s-SWCNT films. To reduce the P3DDT molecules and to obtain a high-purity s-SWCNT solution, we applied a simple heat-assisted polymer removing step. Specifically, by applying a thermal treatment to the solution, the s-SWCNTs are gradually agglomerated and precipitated at the bottom of the solution as shown in Figure 1b (step 3). It is speculated that the thermal energy induces fluctuation of the P3DDT side chains which leads to strong attraction between the s-SWCNTs by the van der Waals and π-π interaction [14,26]. After the thermal treatment, the aggregation of the s-SWCNTs occurs and then the precipitated SWCNT was collected. As a result, a significant portion of P3DDT molecules which are bound to the s-SWCNTs are reduced during this process, providing high-purity s-SWCNTs (step 4). Finally, the purified s-SWCNTs were collected by centrifugation and re-dispersed in a pure solvent by sonication (step 5). Note that the re-dispersion for high purity s-SWCNTs was not influenced by the reduction of the P3DDT molecules (Figure 1b). Figure 1c shows the light absorption spectra of s-SWCNT solutions purified with or without the heat-assisted polymer removing or heat-assisted purification (HAP) step. The light absorption spectra are mainly composed of two s-optical transitions (S11 and S22) corresponding to s-SWCNTs (λ = 605–1500 nm), one m-optical transition (M11) corresponding to m-SWCNTs (λ = 540–605 nm), and a characteristic peak corresponding to P3DDT (λ = 400–520 nm). The results show that in both solutions, the m-SWCNTs and SWCNT bundles are effectively reduced during the purification. More importantly, it was found that the peak corresponding to P3DDT was substantially decreased with the HAP process, indicating that a large portion of P3DDT was reduced from the s-SWCNTs during the heat treatment. It is to note that sufficient thermal energy is required to agglomerate the s-SWCNTs and detach the P3DDT molecules (Figure S2). To find an appropriate temperature range, Raman spectroscopy was carried out for s-SWCNTs which are processed at different temperatures (Figure 1d). From the Raman spectra, it was observed that the characteristic peak associated with P3DDT (1450 cm^−1^) was noticeably reduced when the temperature was 140 and 80 °C. However, despite the decrease of the P3DDT at a lower temperature (80 °C), the thermal energy is not sufficient to separate the P3DDT. This indicates that a sufficient thermal energy is required to detach the P3DDT molecules from the s-SWCNTs. At an optimal temperature (140 °C) for the aggregation of the s-SWCNTs, the side chain melting transition of the P3DDT polymer may occur and then allow both s-SWCNT interactions and aggregation simultaneously [22]. However, despite the optimal temperature for high purity s-SWCNTs proposed by our experimental results in this study, the corresponding correlation is accurately not addressed, which will be future work.

**Figure 2:**
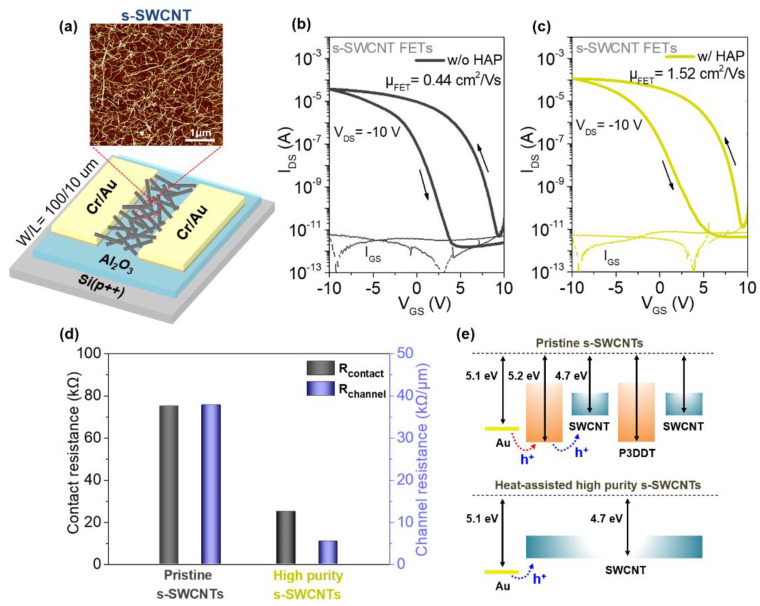
(**a**) A schematic of the SWCNT FET device and an AFM image of the s-SWCNT network. The transfer characteristics of the SWCNT FETs (**b**) without and (**c**) with the HAP process, respectively (W/L = 100/10 μm, V_DS_ = −10 V). (**d**) Comparison of contact and channel resistance for the pristine (w/o the HAP) and high-purity s-SWCNT FETs. (**e**) Energy band diagrams of the s-SWCNT indicating the possible mechanism for the enhancement of charge transport by the HAP process.

To determine the effects of heat-assisted purification of s-SWCNTs on their electrical properties, bottom-gate/bottom-contact structure FETs were fabricated. As channel layers, s-SWCNTs obtained with and without the HAP process were used. Here, Al_2_O_3_ was used as a gate dielectric layer and the channel width (W) and length (L) of the FETs were 100 and 10 μm, respectively (Figure 2a). From the atomic force microscopy (AFM) analysis, it was confirmed that the s-SWCNT film had a uniform two-dimensional network structure with root-mean-square roughness of approximately 1.01 nm. The areal density of s-SWCNTs was >20 nanotubes per μm^2^. Figure 2b,c shows the transfer characteristics of SWCNT FETs without and with the HAP process, respectively. The HAP-processed SWCNT FETs exhibited saturation field-effect mobility of 1.52 cm^2^·V^−1^·s^−1^ with a current on/off ratio of ~10^6^, while, without the HAP process, the FETs showed reduced field-effect mobility of 0.44 cm^2^·V^−1^·s^−1^ (Figure 2b and Figure S3). The improved mobility in HAP-processed SWCNT FETs can be attributed to the substantial reduction of P3DDT bound to s-SWCNTs which can act as the charge blocking elements within the nanotube network as well as at the contacts between the source/drain electrodes and the s-SWCNT channel. Figure 2d shows the variations of contact resistance (R_contact_) and the channel resistance (R_channel_) by the HAP process. Here, resistance values were extracted using the transmission line method (TLM) where the total resistance (R_total_) is defined as R_total_ = R_channel_ + 2R_contact_ (Figure S4). As shown in Figure 2d, both the R_contact_ and R_channel_ decreased from 75 to 25 kΩ and from 37.8 to 5.5 kΩ·μm^−1^ with the HAP process, respectively, indicating that the reduction of P3DDT had significant effects on improving the charge transport in SWCNT FETs. Figure 2e illustrates the possible mechanism for the enhancement of charge transport. With the P3DDT attached to the s-SWCNTs, the hole injection from the Au electrode to the s-SWCNTs can be hindered by an energy barrier of ~0.1 eV. Additionally, between the s-SWCNTs, an energy barrier of ~0.5 eV is formed. In addition, the side chains of P3DDT are electrically insulating which may inhibit efficient hole transport between the s-SWCNTs. In contrast, without the P3DDT, the hole injection from Au electrode to s-SWCNTs can occur without an energy barrier, and also, the hole transport in the s-SWCNT network can be enhanced. However, despite the notable increase in the field-effect mobility, significantly large hysteresis was still observed in the transfer characteristics. Particularly, the HAP-processed SWCNT FETs exhibited hysteresis of 6.8 V which was comparably larger than that without the HAP process (6.0 V). In CNT-based FETs, the hysteresis behavior is attributed to the interfacial charge traps present at the CNT/gate dielectric interface and the adsorption of water molecules on the SWCNT surface [27,28,29,30]. Therefore, the slightly larger hysteresis in HAP-processed SWCNT FETs can be explained by more adsorption of water molecules on the s-SWCNT surface with less amount of P3DDT attached.

The structure of semiconductor/gate dielectric interface has a great influence on the electrical performance of SWCNT FETs, particularly with the staggered bottom-gate structure [31,32,33]. This is mainly due to the stacking structure of the SWCNTs where the nanotubes are overlapped with each other. As a result, nano-gaps can be formed between the SWCNTs and the bottom gate dielectric layer, causing a weak gate-field applied to the SWCNTs [34]. Meanwhile, using top-gate structure and soft gate dielectrics such as polymers and ion-gels, more intimate contacts between SWCNTs and gate dielectric can be made by conformally surrounding the SWCNTs with the soft gate dielectric [35,36,37].

**Figure 3:**
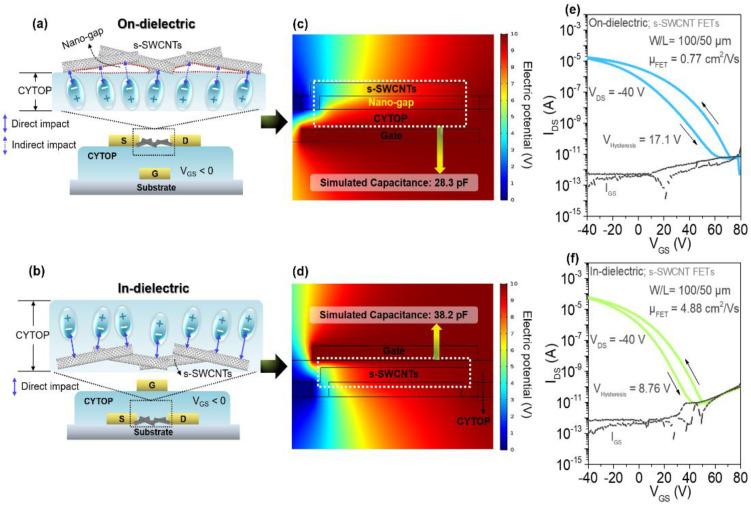
Schematics of SWCNT FETs with CYTOP (**a**) on-dielectric and (**b**) in-dielectric configuration. Corresponding charge accumulation data obtained from the FEA simulation of (**c**) on-dielectric and (**d**) in-dielectric configuration. The transfer characteristics of the SWCNT FETs with (**e**) on-dielectric and (**f**) in-dielectric configuration (W/L = 1000/50 μm, V_DS_ = −40 V).

Accordingly, compared to the staggered bottom-gate structure, nano-gaps may be relatively suppressed by a structural feature of the top-gate structure. To evaluate the influence of using a soft gate dielectric on the electrical performance, both bottom-gate and top-gate structured s-SWCNT FETs were fabricated as shown in Figure 3a,d using CYTOP as a gate dielectric, respectively. Here, the bottom-gate and top-gate FETs were designated as ‘on-dielectric’ and ‘in-dielectric’ structures depending on the position of SWCNTs in respect to the gate dielectric layer. Note that s-SWCNTs with the HAP treatment was used for this test. As shown in Figure 3a, in the case of on-dielectric FETs, nano-gaps with the length of ~50 nm which is estimated by the AFM data exist between the SWCNTs and the gate dielectric, resulting in a weak gate-field and reduced charge accumulation. On the other hand, with the in-dielectric structure (Figure 3d), the CYTOP gate dielectric conformably covers the SWCNTs and more effective charge accumulation can be possible. To verify the effect of FET structure on the charge accumulation, electrical simulations using the finite-element analysis (FEA) method were performed as shown in Figure 3b,e (also, Table S1). It was found that the top-gated in-dielectric device showed a higher capacitance (38.2 pF) compared to bottom-gated on-dielectric devices (28.3 pF) by the suppression of the nano-gaps. Following, SWCNT FETs with on-dielectric and in-dielectric structures were fabricated and their transfer characteristics were analyzed (Figure S5). As shown in Figure 3c,f, the in-dielectric structure FETs exhibited higher average field-effect mobility of 4.07 (standard deviation of 0.65 cm^2^·V^−1^·s^−1^ ), and reduced hysteresis behavior (~8.76 V), which can be attributed to the higher number of holes accumulated in the s-SWCNT channel and the reduction of surface trap states caused by the air exposure.

**Figure 4:**
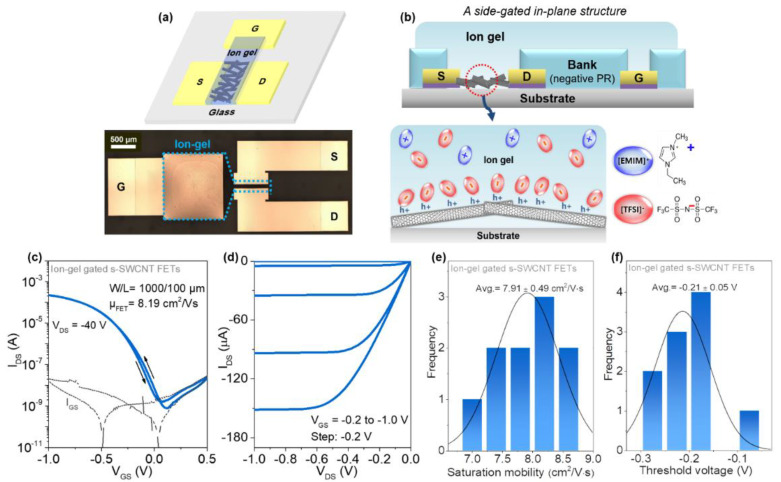
(**a**) Schematic of a side gate high-purity SWCNT FET using ion-gel gate dielectric layer and the optical image of the device (bottom). (**b**) The side view of ion-gel gated SWCNT FET with the channel accumulation by ions in the ion-gel gate dielectric and a chemical structure of EMIM-TFSI. The electrical characteristics: (**c**) a transfer (W/L = 1000/100 μm, V_DS_ = −0.5 V) and (**d**) output curves of ion-gel gated SWCNT FET. Statistics data for (**e**) saturation mobility and (**f**) threshold voltage.

To further improve the electrical properties of SWCNT FETs and to realize a low-voltage operation, high-k ion-gel film was employed as a gate dielectric. Since the ion-gel gate dielectric can be fabricated at a low-temperature using a solution process similar to the CYTOP gate dielectric, SWCNT FETs with a top-gate-like structure can be fabricated as shown in Figure 4a. In particular, after depositing the Cr/Au source/drain and gate electrodes, HAP-processed s-SWCNTs were spin-coated as a channel layer. Then, a negative photoresist was coated and patterned as a bank to isolate the ion-gel gate dielectric. Finally, an ion-gel solution consisting of 1-ethyl-3-methylimidazolium bis(trifluoro-methylsulfonyl) imide ([EMIM][TFSI]), polyethylene glycol diacrylate, and 2-hydroxy-2-methylpropiophenone was drop-casted at the bank region and photo-polymerized by UV exposure (Figure 4b). When a negative gate voltage is applied, an electrical double layer (EDL) is formed at the s-SWCNT/ion-gel interface, inducing accumulation of positive hole charges in the s-SWCNT channel. Figure 4c–f shows the transfer and output characteristics of the ion-gel-gated SWCNT FETs, respectively. The devices exhibited the improved electrical properties such as field-effect mobility of 8.19 cm^2^·V^−1^·s^−1^ and current on/off ratio of ~10^5^ and a low threshold voltage (V_th_) of −0.2 V, attributing to the high capacitance of the ion-gel gate dielectric as well as the conformal contact between the ion-gel and the s-SWCNTs (Figure 4).

**Figure 5:**
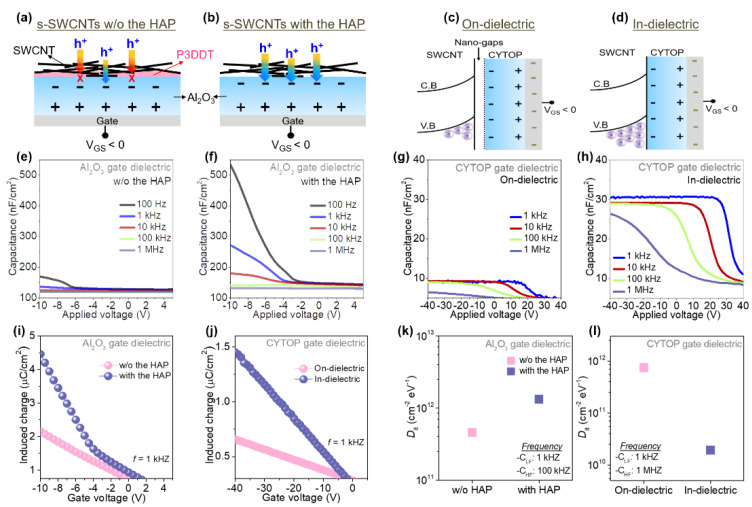
Schematics of the mechanism for the accumulation of hole-carriers in the s-SWCNT (**a**) w/o and (**b**) with the HAP treatment. The energy band diagram models of CYTOP gate (**c**) on- and (**d**) in-dielectric configuration. The C-V characteristics for Al_2_O_3_ gate dielectric with (**e**) w/o and (**f**) with the HAP treatment, and CYTOP gate dielectric with (**g**) on- and (**h**) in-dielectric configuration. Induced charge density of the s-SWCNT FETs with (**i**) Al_2_O_3_ and (**j**) CYTOP gate dielectric. Interface trap density of the s-SWCNT FETs depending on (**k**) the HAP process and (**l**) device configuration.

To further explain the variations of hole accumulation and transport properties depending on the HAP process and the device structure (on- or in-dielectric) with the gate dielectric materials (Al_2_O_3_, CYTOP, or ion-gel), schematics of the mechanism of gate modulation on hole-charge carriers in the s-SWCNT networks and the corresponding energy band diagram models were constructed as shown in Figure 5a–d. Firstly, in the case of the SWCNT FETs on Al_2_O_3_ gate dielectric without the HAP treatment, hole-carriers can be accumulated near the Al_2_O_3_/s-SWCNT interface under a negative gate bias (V_GS_ < 0) to form a current pathway. As aforementioned, however, the charge carrier transport would be interrupted by the P3DDT polymer bound to s-SWCNTs which plays a role as a block-barrier for hole carriers. Additionally, the P3DDT may prevent gate-field from being induced charges at the interface, resulting in a small number of accumulating hole-carriers (Figure 5a). On the other hand, as shown in Figure 5b, the s-SWCNTs with the HAP treatment could lead to a number of charges induced by gate-field as well as enhance the charge-carrier transport due to the reduced P3DDT. However, it is noteworthy that compared to the s-SWCNT without the HAP treatment, a larger area of the s-SWCNT channel layer would be exposed to ambient gases by the reduction of P3DDT, causing a large hysteresis behavior, which is in agreement with the transfer curve of s-SWCNT TFTs with the HAP process (Figure 2c). In addition to the effect of the HAP process on the electrical characteristics of the SWCNT FETs, device configurations significantly influence the entire properties of the TFTs. Figure 5c and d shows the comparison of CYTOP on- and in-dielectric configurations for the accumulation of hole-carriers, respectively. In the case of CYTOP gate dielectric and on-dielectric device structure, nano-gaps exist between the s-SWCNT channel and the CYTOP as shown in Figure 5c, causing reduced gate-field in the s-SWCNT channel and smaller number of accumulated holes. With the in-dielectric structure, however, the formation of the nano-gaps is suppressed by the conformal contact made between the CYTOP gate dielectric and the s-SWCNTs (Figure 5d). Subsequently, more numbers of holes are accumulated in the channel layer resulting in improved field-effect mobility. Similarly, with the ion-gel gate dielectric and in-dielectric device structure (a side-gated in-plane structure), cations migrated to the s-SWCNT/ion-gel interface under a negative gate bias and relatively high number of holes can be accumulated in the s-SWCNT channel even at a low gate bias (Figure S6a).

To verify the above the mechanisms and energy band models, capacitance-voltage (C-V) characteristics of metal-insulator-semiconductor (MIS) devices with Au/(Al_2_O_3_, CYTOP or ion-gel)/s-SWCNTs structures were analyzed. As shown in Figure 5e–h, in all cases, the number of accumulated hole carriers increased with negative gate biasing. Additionally, the capacitance value was significantly decreased with the applied frequency. The increase of capacitance at low frequency is attributed to the interface states that cannot respond quickly. In contrary, at the low frequency, the charges can follow the ac signal easily and the interface state capacitance can be considered to the total capacitance. The C-V characteristics were largely varied with the presence of P3DDT in the s-SWCNT channel. Particularly, with the HAP-processed s-SWCNTs, higher capacitance value was obtained compared to the s-SWCNTs with P3DDT (Figure 5e,f). As described, compared to the pristine s-SWCNTs without the HAP treatment, the s-SWCNTs with the HAP treatment could be exposed to air through the reduced P3DDT bound to s-SWCNT, resulting in higher area capacitance. This result is due to an increase in the effective contact area between s-SWCNTs and gate dielectric. However, despite such positive features, the reduction of the P3DDT would negatively affect a hysteresis of the SWCNT FETs due to the direct adsorption of water molecules on the s-SWCNT surfaces, which is in tune with the hysteresis characteristic of HAP-processed SWCNT FETs. In addition, the device configuration has a significant impact on the C-V characteristics. As shown in Figure 5g,h, with the in-dielectric FET structure completely covering the s-SWCNTs with the CYTOP gate dielectric, the capacitance value was increased in all frequency domains compared to the on-dielectric device, supporting the elimination of the nano-gaps. Additionally, the ion-gel gated s-SWCNTs showed a similar trend to the in-dielectric structure and the highest areal capacitance value (~50 µF·cm^−2^) due to a high-k ion-gel film (Figure S6b,c).

Additionally, from the C-V characteristics, we extracted the induced charge density (Q_ind_) in the s-SWCNT channel layer using the following Equation (1) [35,38],

Q_ind_ = C_GD_ (V_G_ − V_th_) (1)

where C_GD_ and V_th_ are the areal capacitance of gate dielectric and the threshold voltage in the linear region, respectively. As a result, both the HAP process reducing the P3DDT polymer, and the in-dielectric device structure providing the conformal contact at the interface improved the Q_ind_, resulting in a lot of the accumulated hole carriers and the enhanced charge transport of the s-SWCNT (Figure 5i,k). Additionally, for further analysis of the interface between the s-SWCNT and gate dielectric, the interface trap density (D_it_) has been investigated from the C-V characteristics using the Equation (2) below [38,39],

(2) $$D_{\mathrm{it}}=\frac{C_{\mathrm{LF}}-C_{\mathrm{HF}}}{q\left(1-\frac{C_{\mathrm{LF}}}{C_{\mathrm{di}}}\right)\left(1-\frac{C_{\mathrm{HF}}}{C_{\mathrm{di}}}\right)\mathrm{WL}}$$

where C_LF_, C_HF_, C_di_, and q are the low-frequency, high-frequency, gate dielectric capacitance, and electron charge, respectively. The results indicate that the interface trap density considerably increased as the P3DDT polymers bonded with the s-SWCNTs were reduced by the HAP process, as shown in Figure 5j. It means that a clean s-SWCNT surface with the reduced P3DDT can lead to active adsorption of water molecules, causing a larger hysteresis behavior. Therefore, it is important to reduce D_it_, which degrades electrical performance. Figure 5i shows the comparison of the D_it_ depending on device configuration (on- and in-dielectric). As expected, with the in-dielectric configuration covering the entire s-SWCNT channel layer, the D_it_ decreased considerably. This result is attributed to both a conformal/imitate contact mitigating nano-gaps that can interrupt inducible charges at the interface and the passivation effect that can prevent the absorption of ambient molecules on the s-SWCNT channel surface. Similarly, for the ion-gel gated s-SWCNTs FETs, the interface trap density would be positively relieved due to more conformal contact with an ion-gel dielectric.

## 4. Conclusions

In summary, we achieved high purity semiconducting s-SWCNT solution by a facile heat-assisted purification (HAP). Through Raman analysis and absorption spectroscopy, it confirmed that conjugated P3DDT polymer wrapped s-SWCNT was successfully decreased after the HAP process. In addition, in order to investigate the effect of the reduction of the P3DDT on the electrical characteristics, we fabricated s-SWCNT FETs on an ALD-Al_2_O_3_/Si substrate using the HAP-processed s-SWCNT solution. As a result, the HAP-processed s-SWCNT FETs exhibited improved electrical performances due to the considerable reduction of P3DDT interfering with the charge transport within the SWCNT network. However, a large hysteresis was still present as well as it was more deteriorated than that without the HAP process. This result would be attributed to the adsorption of water molecules on the clean SWCNT surface with less P3DDT attached, causing a large interface trap density. Furthermore, by comparing the induced charge and interface trap densities extracted from finite-element-analysis (FEA) computer simulation and the C-V characteristics of the on-dielectric and in-dielectric configuration of s-SWCNT FETs simultaneously, it was founded that conformal contact between the gate dielectric and the SWCNT network not only provides a higher gate-field effect to the SWCNT layer, but also lowers the interface trap density. Finally, to ensure the viability of the more favorable gate-field-induced semiconducting surface behaviors of s-SWCNT, we implemented conformally gated highly capacitive s-SWCNT FETs with ion-gel dielectrics, demonstrating field-effect mobility of ~8.19 cm^2^/V⋅s and on/off current ration of ~10^5^ along with negligible hysteresis. We believe that the understanding the configuration of the s-SWCNT FETs is crucial to achieve a facile route to high-performance and stable solution-processed semiconductor devices with marginal complexity, offering compatibility with standard CMOS processing and large-scaled on-chip device applications.

## Data Availability

The data presented in this study are available on request from the corresponding author.

## Supplementary Materials

The following are available online at https://www.mdpi.com/article/10.3390/ma14123361/s1, Figure S1: Capacitance per area-frequency (*C–F*) characteristics of (**a**) Al_2_O_3_; (**b**) CYTOP, and (**c**) ion-gel gate dielectric layers using a metal/insulator/metal (MIM; Au/Al_2_O_3_, CYTOP, or ion-gel/Au) structure, Figure S2: Optical image of heat-assisted s-SWCNT solutions with different temperature of 80, 100, 120, and 140 °C. The aggregation of the s-SWCNTs increases with increasing temperature, Figure S3: Statistical distribution of (**a**,**c**) saturation mobility and (**b**,**d**) threshold voltage (V_TH_) of the s-SWCNT FETs on Al_2_O_3_ gate dielectric layer without or with the HAP treatment, Figure S4: Total resistance (R_total_) extracted by transmission line method (TLM) of the s-SWCNT FETs (**a**) without and (**b**) with the HAP treatment. The insets are the energy band diagrams of the s-SWCNTs, Figure S5: Statistical distribution of (**a**,**d**) saturation mobility, (**b**,**e**) threshold voltage (V_TH_), and (**c**,**f**) hysteresis for the s-SWCNT FETs on CYTOP gate dielectric with different device configurations: on- and in-dielectric configuration, Figure S6: (**a**) An energy band diagram model of a side gate high-purity SWCNT FET using ion-gel gate dielectric layer. (**b**) The *C–V* characteristic and (**c**) induced charge density of ion-gel gated s-SWCNT FETs, Table S1: Properties of the materials used for COMSOL simulation.
