# Supplementary material for: Conformally Gated Surface Conducting Behaviors of Single-Walled Carbon Nanotube Thin-Film-Transistors
Source: Materials (Basel). 2021 Jun 17;14(12):3361. doi: 10.3390/ma14123361 (PMC8234559; doi:10.3390/ma14123361)
Supplement: Supplementary file 1 [file materials-14-03361-s001.zip › materials-1237830-supplementary.pdf]

materials

Supplementary Materials

Conformally Gated Surface Conducting Behaviors of Single-Walled Carbon Nanotube Thin-Film-Transistors

Kyung-Tae Kim [1,†], Keon Woo Lee [1,†], Sanghee Moon [1], Joon Bee Park [1], Chan-Yong Park [1], Seung-Ji Nam [1], Jaehyun Kim [2], Myoung-Jae Lee [3], Jae Sang Heo [4,*] and Sung Kyu Park [1,*]

[1] Department of Electrical and Electronics Engineering, Chung-Ang University, Seoul 06974, Korea; ktkim0314@gmail.com (K.T.K.); lkw9941@naver.com (K.W.L.); mshhwaa@gmail.com (S.M.); wnsbee@gmail.com (J.B.P.); mgs01170@gmail.com (C.-Y.P.); seungjieee@gmail.com (S.-J.N.);
[2] Department of Chemistry and Materials Research Center, Northwestern University, 2145 Sheridan Road, Evanston, IL 60208, USA; jaehyun@northwestern.edu
[3] Convergence Research Institute, Daegu Gyeongbuk Institute of Science and Technology (DGIST), Daegu 42988, Korea; myoungjae.lee@dgist.ac.kr (M.J.L.)
[4] School of Advanced Materials Science and Engineering, Sungkyunkwan University, Suwon 16419, Korea.
† These authors contributed equally to this work.
* Correspondence: heojs38@gmail.com (J.S.H.); skpark@cau.ac.kr (S.K.P.)

This "Supporting Information" includes: Figure S1–S6
Table S1

Citation: Kim, K.-T.; Lee, K.W.; Moon, S.; Park, J.B.; Park, C.-Y.; Nam, S.-J.; Kim, J.; Lee, M.-J.; Heo, J.S.; Park, S.K. Conformally Gated Surface Conducting Behaviors of Single-Walled Carbon Nanotube Thin-Film-Transistors. *Materials* **2021**, *14*, 3361. https://doi.org/10.3390/ma14123361

Academic Editor: Antonio Di Bartolomeo, Chang-Hyun Kim, Pedro Barquinha

Received: 12 May 2021
Accepted: 11 June 2021
Published: 17 June 2021

Publisher's Note: MDPI stays neutral with regard to jurisdictional claims in published maps and institutional affiliations.

Copyright: © 2021 by the authors. Licensee MDPI, Basel, Switzerland. This article is an open access article distributed under the terms and conditions of the Creative Commons Attribution (CC BY) license (http://creativecommons.org/licenses/by/4.0/).

1. Electrical Characterization (*C-F*) of Gate Dielectric Layers.

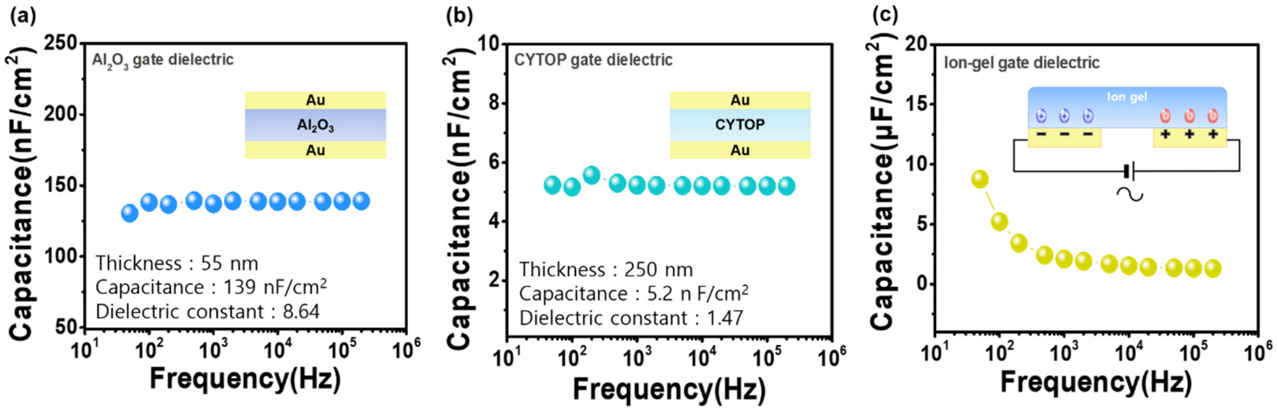

Figure S1. Capacitance per area-frequency (*C*-*F*) characteristics of (**a**) Al_2O_3; (**b**) CYTOP, and (**c**) ion-gel gate dielectric layers using a metal/insulator/metal (MIM; Au/Al_2O_3, CYTOP, or ion-gel/Au) structure.

2. Heat-Assisted Purification (HAP) of s-SWCNT Solution.

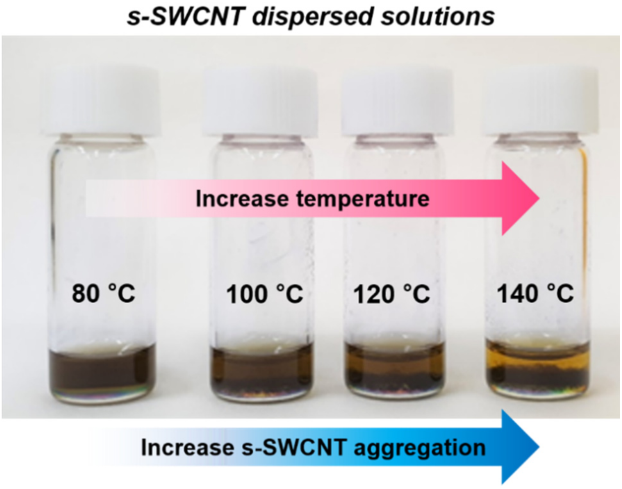

Figure S2. Optical image of heat-assisted s-SWCNT solutions with different temperature of 80, 100, 120, and 140 °C. The aggregation of the s-SWCNTs increases with increasing temperature.

3. The Statistical Data of Electrical Performances for the s-SWCNT FETs on Al_2O_3 Gate Dielectric.

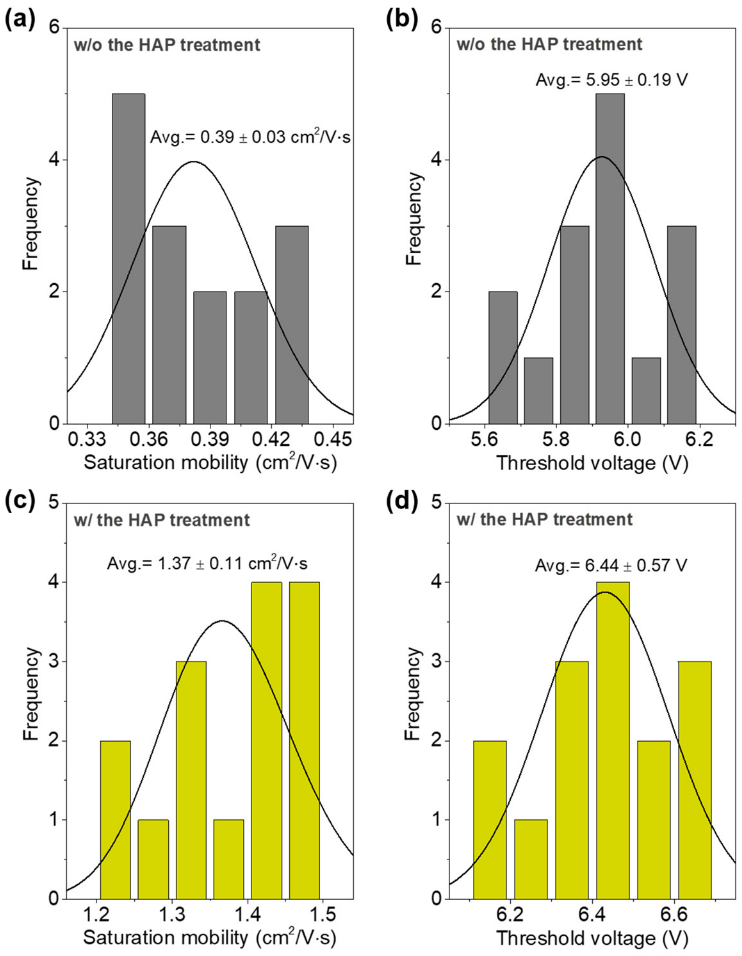

Figure S3. Statistical distribution of (**a**,**c**) saturation mobility and (**b**,**d**) threshold voltage (V_{TH}) of the s-SWCNT FETs on Al_2O_3 gate dielectric layer without or with the HAP treatment.

4. Transmission Line Method (TLM) for Extracting Contact and Channel Resistance Values.

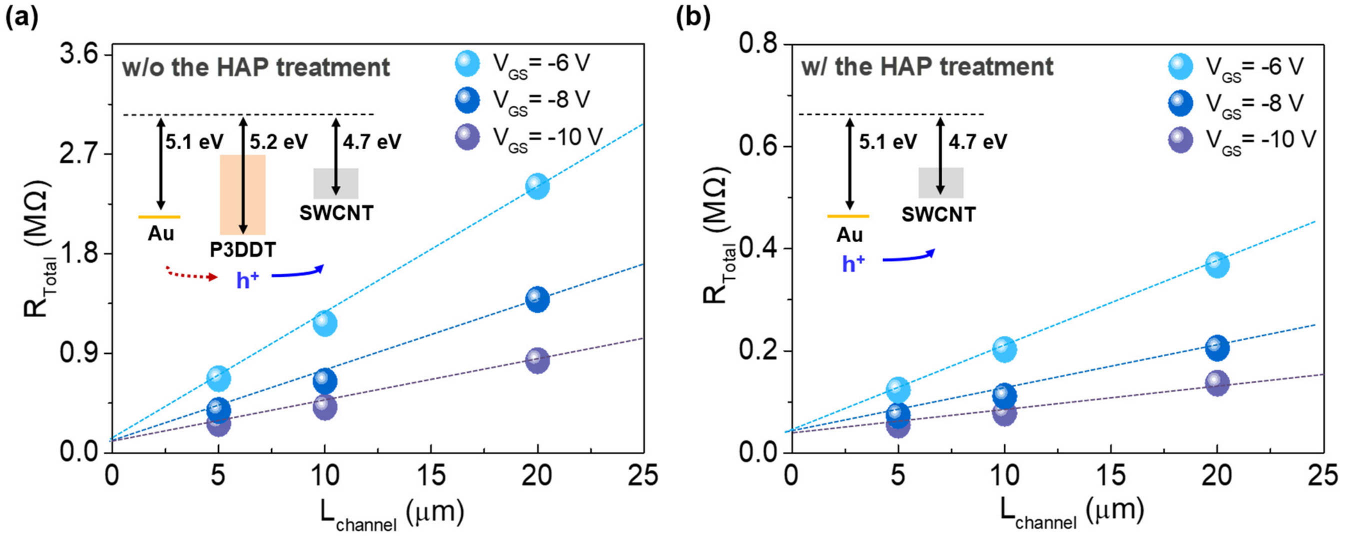

Figure S4. Total resistance (R_{total}) extracted by transmission line method (TLM) of the s-SWCNT FETs (**a**) without and (**b**) with the HAP treatment. The insets are the energy band diagrams of the s-SWCNTs.

5. Statistical Distribution of the s-SWCNT FETs with CYTOP Gate On- and In-Dielectric Configuration.

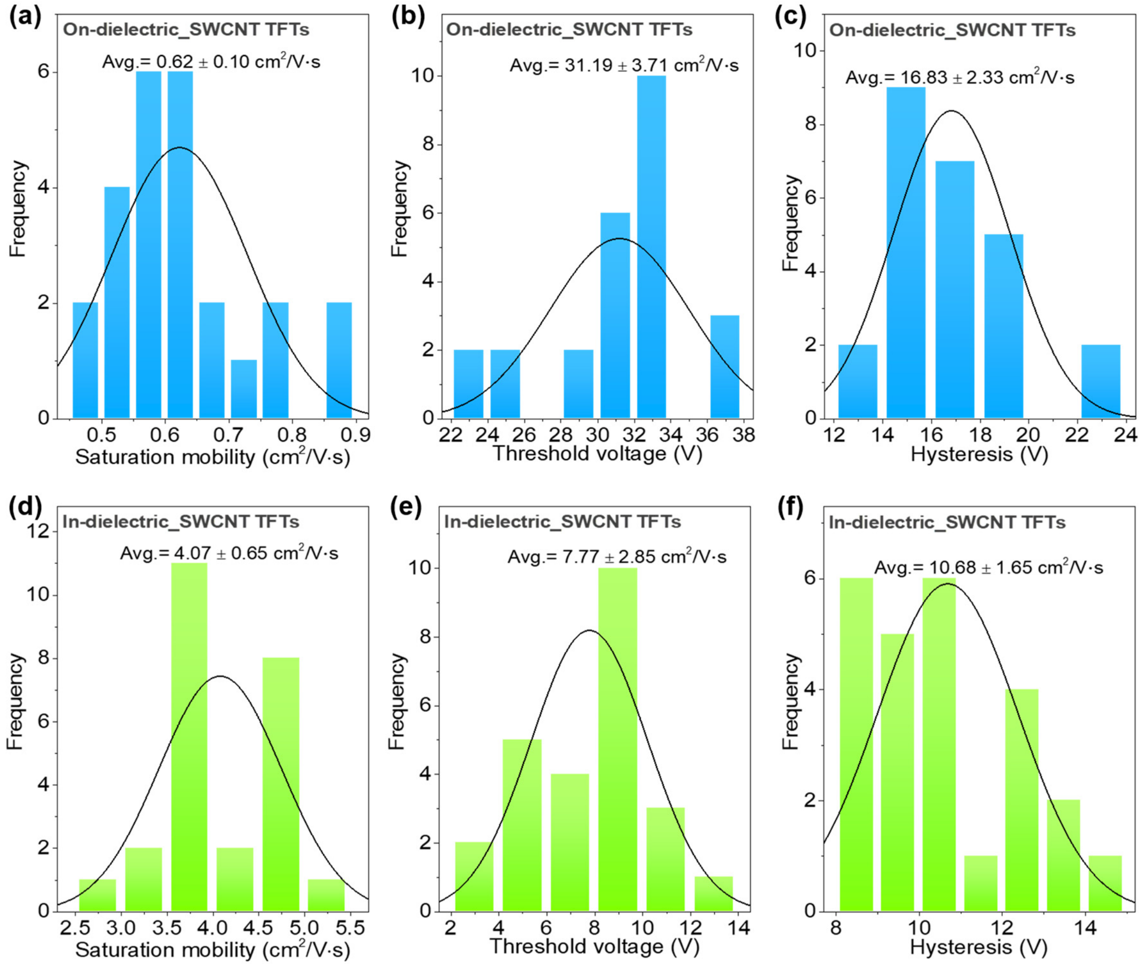

Figure S5. Statistical distribution of (**a**,**d**) saturation mobility, (**b**,**e**) threshold voltage (V_{TH}), and (**c**,**f**) hysteresis for the s-SWCNT FETs on CYTOP gate dielectric with different device configurations: on- and in-dielectric configuration.

6. The ion-gel gate dielectric and in-dielectric device structure.

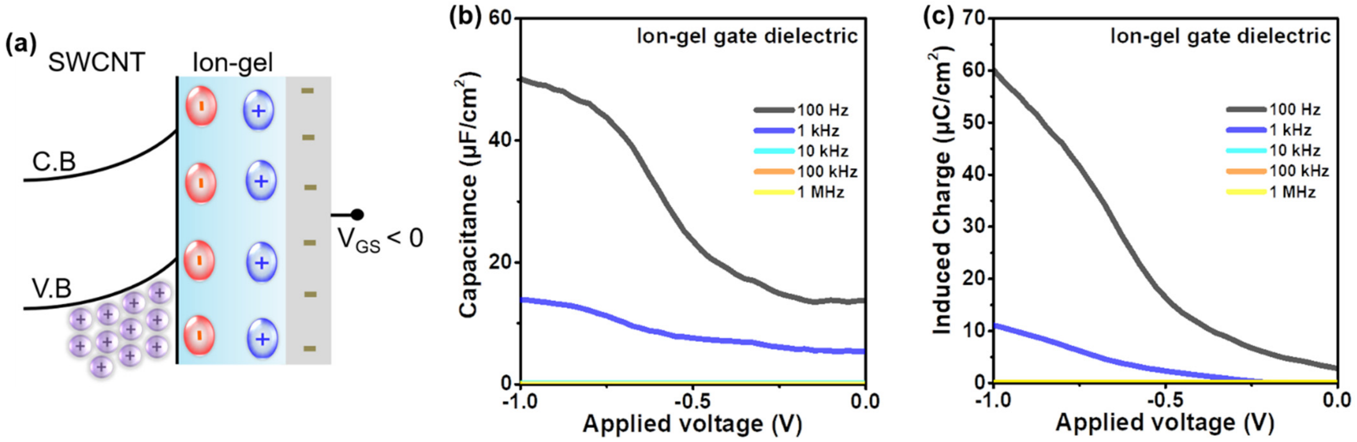

Figure S6. (**a**) An energy band diagram model of a side gate high-purity SWCNT FET using ion-gel gate dielectric layer. (**b**) The *C-V* characteristic and (**c**) induced charge density of ion-gel gated s-SWCNT FETs.

Table S1. Properties of the materials used for COMSOL simulation.

Layer	Material	Thickness (nm)	Relative Permittivity
Gate & Source/Drain	Au	30	1.62
Gate dielectric	CYTOP	250	1.46
Channel	SWCNT	3	73 [1]
Substrate	Glass	200	5
Atmosphere	Air	—	1.00059

References

1. Shuba, M.V.; Paddubskaya, A.G.; Kuzhir, P.P.; Maksimenko, S.A.; Flahaut, E.; Fierro, V.; Celzard, A.; Valusis, G. Short-length carbon nanotubes as building block for high dielectric constant materials in terahertz range. *J. Phys. D. Appl. Phys.* **2017**, *50*, 08LT01.
